# Molecular Detection, Aggressiveness, and Vegetative Compatibility of *Macrophomina phaseolina* Isolates from Common Bean Fields in Sinaloa, Mexico

**DOI:** 10.3390/jof12030218

**Published:** 2026-03-18

**Authors:** Edgar Edel Rodríguez-Palafox, Juan Manuel Tovar-Pedraza, Hugo Beltrán-Peña, Elizabeth García-León, Moisés Camacho-Tapia, Santos Gerardo Leyva-Mir, Alma Rosa Solano-Báez, Guillermo Márquez-Licona

**Affiliations:** 1 Departamento de Ciencias Naturales y Exactas, Universidad Autónoma de Occidente, Unidad Los Mochis, Los Mochis 81223, Sinaloa, Mexico; edgarrodriguez2295@gmail.com; 2 Centro de Investigación en Alimentación y Desarrollo, Subsede Culiacán, Laboratorio de Fitopatología, Culiacán 80110, Sinaloa, Mexico; juan.tovar@ciad.mx; 3 Facultad de Agricultura del Valle del Fuerte, Universidad Autónoma de Sinaloa, Juan José Ríos 81110, Sinaloa, Mexico; hugobeltran@uas.edu.mx; 4 Instituto Nacional de Investigaciones Forestales, Agrícolas y Pecuarias, Campo Experimental Valle del Fuerte, Guasave 81110, Sinaloa, Mexico; garcia.elizabeth@inifap.gob.mx; 5 Departamento de Parasitología Agrícola, Universidad Autónoma Chapingo, Texcoco 56230, Estado de México, Mexico; mcamachot@chapingo.mx (M.C.-T.); lsantos@correo.chapingo.mx (S.G.L.-M.); 6 Centro de Desarrollo de Productos Bióticos, Instituto Politécnico Nacional, Yautepec 62731, Morelos, Mexico; asolanob@ipn.mx

**Keywords:** *Phaseolus vulgaris*, *Macrophomina phaseolina*, charcoal rot, pathogenicity, mycelial compatibility

## Abstract

Charcoal rot of common bean, caused by *Macrophomina*, is one of the most economically important diseases worldwide. In Mexico, charcoal rot of bean has been associated exclusively with *M. phaseolina*; however, in recent years, new *Macrophomina* species affecting various crops have been described globally. Information on this pathogen in common bean in Mexico remains limited. Therefore, the objectives of this study were to characterize *Macrophomina* isolates obtained from bean fields in northern Sinaloa morphologically and molecularly using species-specific primers, and to determine their aggressiveness and vegetative compatibility groups (VCGs). During the 2020–2021 growing season, 50 *Macrophomina* isolates were obtained from common bean tissues exhibiting charcoal rot symptoms collected from 12 fields in the municipalities of Ahome and Guasave, Sinaloa, Mexico. Molecular analysis using species-specific primers for three *Macrophomina* species (*M. phaseolina*, *M. pseudophaseolina*, and *M. euphorbiicola*) identified all 50 isolates as *M. phaseolina*. Pathogenicity tests indicated that the *M. phaseolina* isolates differed in aggressiveness toward common bean plants. Mycelial compatibility assays revealed at least seven vegetative compatibility groups among *M. phaseolina* isolates distributed across northern Sinaloa. To our knowledge, this is the first study to provide phenotypic characterization, aggressiveness assessment, vegetative compatibility grouping, and species-specific primer-based identification of *M. phaseolina* isolates from common bean fields in Sinaloa, Mexico.

## 1. Introduction

Common bean (*Phaseolus vulgaris* L.) is one of the most important crops worldwide. Mexico ranks eighth among the largest global producers of this legume [1], with 71.5% of national production concentrated in five principal states: Zacatecas, Sinaloa, Nayarit, Chiapas, and Durango [2]. Charcoal rot, caused by species of the genus *Macrophomina*, is an economically important disease of common bean worldwide. This disease can occur at nearly all phenological stages, infecting seeds, seedlings, and mature plants [3]. Early infections significantly reduce plant density and, consequently, yield. The pathogen persists as microsclerotia in seed, crop residues, and soil, serving as inoculum for subsequent infection cycles [4,5,6,7]. In bean crops established under low relative humidity, high temperatures, and suboptimal agronomic management, disease incidence can result in yield losses of up to 60% [8,9].

Symptoms of charcoal rot in beans begin at the stem base due to vascular tissue obstruction in the roots and in stem regions adjacent to the soil line. As disease severity increases, symptoms may extend to aerial tissues, including leaves. In seedlings, lesions appear as irregular spots at the base of the cotyledons and progress along the stem, causing damping-off, dry dark rot, and subsequent wilting or plant death [8]. In mature plants, dry dark rot is observed, accompanied by abundant microsclerotia and embedded fruiting bodies (pycnidia) throughout infected tissues. Typical lesions range from gray to black along affected stems and are frequently associated with premature defoliation, reduced vigor, and diminished yield [10].

Accurate phylogenetic species identification is essential for understanding epidemiology and establishing effective management strategies [6,11,12]. Species circumscription within *Macrophomina* has historically relied on morphological traits, host range, and disease symptoms and signs [12]. However, conventional taxonomic characters have proven insufficient for resolving cryptic species within this genus. According to Babu et al. (2010) [13], *Macrophomina phaseolina* is highly heterogeneous in terms of morphology, physiology, ecology, and general characteristics. The genus *Macrophomina* was previously considered monotypic [12]. Nonetheless, molecular phylogenetic analyses have provided improved resolution, enabling the distinction of multiple species [6,11,12,14].

*Macrophomina phaseolina* is the most common species and the type species of the genus. Charcoal rot caused by *M. pseudophaseolina* was first described in cowpea (*Vigna unguiculata*), peanut (*Arachis hypogaea*), roselle (*Hibiscus sabdariffa*), and okra (*Abelmoschus esculentus*) in Senegal [6], and subsequently reported in peanut, cotton (*Gossypium hirsutum*), and castor bean (*Ricinus communis*) in Brazil [11], as well as in lentil (*Lens culinaris*) in Algeria [15]. In 2018, *M. euphorbiicola* was described as causing charcoal rot in physic nut (*Jatropha gossypifolia*) seed in Brazil [11]. Later, *M. tecta* was reported as a potential pathogen of maize (*Zea mays*) in Argentina, and, in Australia, affecting sorghum (*Sorghum bicolor*) and mung bean (*Vigna radiata*) [16], as well as sesame (*Sesamum indicum*) in India [17]. In contrast, *M. vaccinii* has been reported only in China, infecting *Pogostemon cablin* and *Vaccinium* spp. The restricted host range of certain *Macrophomina* species, together with interspecific variability, underscores the need for further studies to investigate potential host specialization, intraspecific genetic diversity, and pathogenic variability [18,19,20].

Currently, species delimitation within *Macrophomina* is based on multilocus phylogenetic analyses, including the internal transcribed spacer (ITS) region and partial sequences of the actin (*act*), β-tubulin (*tub2*), calmodulin (*cal*), and translation elongation factor 1-alpha (*tef1-α*) genes [6,11]. Additionally, the use of species-specific primers in conventional polymerase chain reaction (PCR) assays has proven to provide accurate, reproducible, and rapid species identification and detection [14,21,22]. In Mexico, charcoal rot of common bean has been attributed exclusively to *M. phaseolina* [23,24]; however, no studies have been conducted using isolates from fields in Sinaloa. Therefore, the objective of this study was to determine the diversity of *Macrophomina* species associated with charcoal rot of common bean in northern Sinaloa using species-specific primers and to assess the aggressiveness and vegetative compatibility of the recovered fungal isolates.

## 2. Materials and Methods

### 2.1. Sample Collection

From November 2020 to January 2021, surveys were conducted in 12 commercial fields of common bean cv. Azufrado Higuera in northern Sinaloa (Table 1).

**Figure 1:**
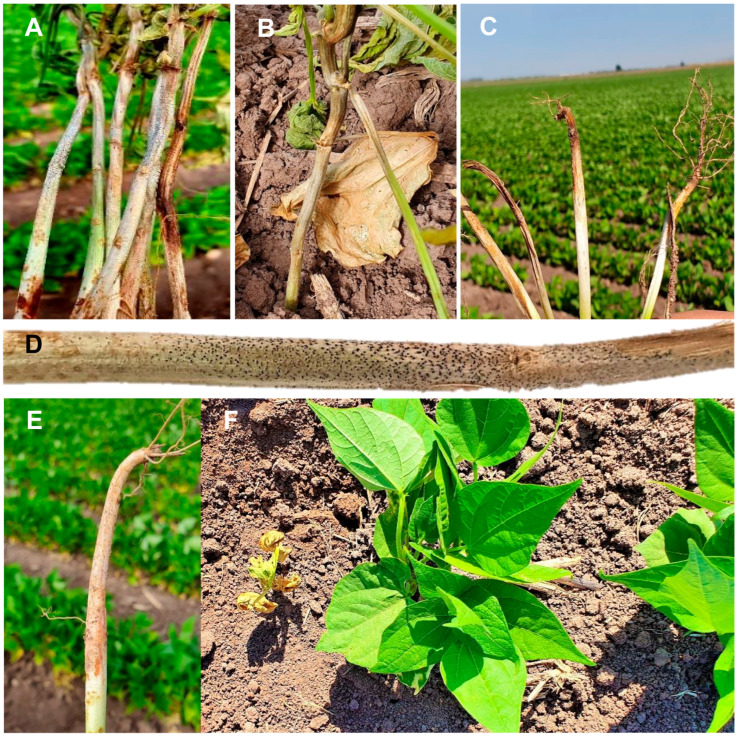
Common bean plants cv. Azufrado Higuera showing symptoms of charcoal rot. (**A**,**B**) Gray to dark brown dry rots with microsclerotia embedded in the stem and leaf wilting. (**C**) Stem base showing girdling symptoms and roots with dry rot, necrosis, and dark lesions. (**D**) Dry stem exhibiting microsclerotia. (**E**) Plant with poorly developed root system. (**F**) Symptomatic plant (**left**) and asymptomatic plant (**right**) of the same age.

During each survey, stems and roots exhibiting typical charcoal rot symptoms and signs were collected (Figure 1). Disease incidence at each of the 12 sampling sites was estimated using the five-of-diamonds sampling pattern. At each point, a subsample of 100 plants was assessed, and all plants exhibiting symptoms consistent with charcoal rot were recorded, yielding a total of 500 plants evaluated per site.

### 2.2. Isolation, Purification, and Preservation of Isolates

*Macrophomina* isolates were obtained following the methodology described by Leyva-Mir et al. (2015) [25], with minor modifications. Fragments (5 × 5 mm) were excised from the margin of lesions, surface-disinfested in 1% sodium hypochlorite (NaClO) for 2 min, rinsed three times with sterile distilled water, and dried on sterile absorbent paper. Disinfested tissue fragments were plated onto potato dextrose agar (PDA; Difco, Sparks, MD, USA) in Petri dishes and incubated at 25 °C in continuous darkness for four days in an incubator (Terlab, Guadalajara, Jalisco, Mexico). Colonies exhibiting typical characteristics of the genus *Macrophomina* were transferred to fresh PDA plates. Monohyphal cultures were obtained by transferring hyphal tips developed on water agar (WA) to PDA plates. The *Macrophomina* isolates used in this study were deposited in the Culture Collection of Phytopathogenic Fungi of the Facultad de Agricultura del Valle del Fuerte, Universidad Autónoma de Sinaloa (Juan José Ríos, Ahome, Sinaloa, Mexico) under accession numbers FAVF274–FAVF323 (Table 1). Cultures were preserved as mycelial plugs in 15% glycerol at −20 °C, in sterile distilled water at 4 °C, and in mineral oil at room temperature.

### 2.3. Morphological and Cultural Characterization

Macroscopic and microscopic characteristics were examined for 50 *Macrophomina* isolates obtained from symptomatic bean plants. Mycelial growth rate was determined as follows: mycelial plugs (6 mm diameter) taken from the actively growing margin of four-day-old colonies were transferred to PDA and incubated at 25 °C in continuous darkness. Colony diameter was measured perpendicularly at 24 h intervals for 4 days using a digital caliper (Truper, Jilotepec de Molina Enríquez, Estado de México, Mexico). Three replicates were evaluated per isolate. Mycelial diameter data were used to calculate growth rate according to Zervakis et al. (2001) [26]. Four-day-old colonies were visually characterized based on colony color (surface and reverse) using the color charts of Rayner (1970) [27]. Colony pigmentation, mycelial type, and microsclerotia masses were recorded using a biological microscope (Leica, Wetzlar, Germany). The entire experiment was conducted twice.

### 2.4. Detection Using Species-Specific Primers

Total genomic DNA was extracted from seven-day-old *Macrophomina* colonies grown on PDA. Mycelium from each isolate was scraped with a sterile slide, ground in a sterile mortar with liquid nitrogen, and transferred to a 1.5 mL microcentrifuge tube containing 500 µL extraction buffer (100 mM Tris-HCl pH 8, 20 mM EDTA pH 8, 1.4 M NaCl, 3% CTAB, 0.2 mg mL^−1^ proteinase K). Samples were vortexed for 10 s and incubated at 65 °C for 60 min. Subsequently, 700 µL chloroform: isoamyl alcohol (24:1, *v*/*v*) was added, vortexed for 10 s, and centrifuged at 13,000× *g* for 10 min in a 5810 R centrifuge (Eppendorf, Framingham, MA, USA). The supernatant was transferred to a new tube, mixed with 700 µL isopropanol, inverted gently, and incubated at −20 °C for 10 min. After centrifugation at 13,000× *g* for 10 min, the supernatant was discarded. The pellet was washed with 500 µL of 70% ethanol, centrifuged at 13,000× *g* for 5 min, and air-dried. DNA was resuspended in 100 µL nuclease-free sterile water. DNA quality and concentration were quantified using a Q 3000 UV spectrophotometer (Quawell, San Jose, CA, USA), and samples were stored at −20 °C until use. Polymerase chain reaction (PCR) assays were performed using species-specific primers described by Santos et al. (2020) [14], targeting the translation elongation factor 1-alpha (*tef1-α*) gene for *Macrophomina phaseolina*, *M. pseudophaseolina*, and *M. euphorbiicola*. PCRs were prepared in a final volume of 25 µL containing 1× PCR buffer, 2.5 mM MgCl_2_, 0.2 mM dNTPs, 0.4 µM of each primer, 0.04 U DNA polymerase (Promega, Madison, WI, USA), and 4 ng template DNA. Amplification was performed in a C-1000 thermocycler (Bio-Rad, Hercules, CA, USA) under the following conditions: initial denaturation at 95 °C for 3 min; 35 cycles of 95 °C for 30 s, 67 °C for 30 s, and 72 °C for 1 min; and a final extension at 72 °C for 10 min. Annealing temperatures for MeEFF/MeEFR, MpEFF/MpEFR, and MsEFF/MsEFR were 65, 67, and 69 °C, respectively. PCR products were separated by electrophoresis on 1% agarose gels in 1× TAE buffer at 90 V for 45 min and visualized using a Gel Doc XR+ Gel Documentation System (Bio-Rad, USA).

### 2.5. Vegetative Compatibility

Mycelial compatibility tests for the 50 *Macrophomina* isolates were conducted on 90 mm Petri dishes containing PDA. A 6 mm mycelial plug from a four-day-old actively growing colony of the test isolate was placed at the center of the plate, with five different isolates positioned at the periphery. All possible isolate combinations were evaluated with three replicates. Plates were incubated at 25 °C in continuous darkness, and colony interactions were assessed at 7, 10, and 14 days. Compatibility was determined visually. A positive reaction was recorded when colonies merged without visible demarcation, forming a continuous mycelial mat. A negative reaction was recorded when a clearly defined barrage zone (incompatibility line) formed between colonies, often accompanied by hyphal degradation. The experiment was conducted twice.

### 2.6. Pathogenicity and Aggressiveness Tests

Pathogenicity and aggressiveness of the 50 *Macrophomina* isolates were evaluated on common bean plants cv. Azufrado Higuera. Seeds were washed under running water, surface-disinfested in 1% sodium hypochlorite for 2 min, rinsed three times with sterile distilled water, and dried on sterile absorbent paper. Seeds were sown in sterilized peat moss substrate (PRO-MOSS III, Quakertown, PA, USA) in 1 kg capacity pots, with two seeds per pot and five replicates per isolate. For each isolate, thirty days after sowing, wounds were made at the base of the stems of five plants using a sterile toothpick, and a 6 mm PDA plug with four-day-old mycelial growth was placed over the wound. Ten control plants were inoculated with sterile PDA plugs without fungal growth. After inoculation, all inoculated and control plants were watered to field capacity and covered with plastic bags to maintain a humid environment (RH > 80%). All plants were incubated in the laboratory at 25 °C under a 12:12 light/dark photoperiod for 6 days. After this period, the bags were removed. The plants were then transferred to the greenhouse and maintained at ambient temperature (25–35 °C) for one more week. Lesion length was measured at 12 days post-inoculation using a digital caliper (Truper, Mexico). Isolates were classified into three categories of aggressiveness: low (<20 mm lesion length), moderate (20–30 mm), and high (>30 mm). The experiment followed a randomized complete block design and was conducted twice. Normality of incidence and aggressiveness data was assessed using the Shapiro–Wilk test. Parametric analysis of variance (ANOVA) was performed, and means were compared using Tukey’s test at a significance level of *p* ≤ 0.05. Statistical analyses were conducted using MINITAB 19. Re-isolations were made from the inoculated plants. The recovered fungi were re-identified using specific primers to confirm their identity.

## 3. Results

### 3.1. Disease Incidence

Disease incidence in the 12 common bean fields (cv. Azufrado Higuera) located in the municipalities of Guasave and Ahome in the state of Sinaloa (Mexico), ranged from 4.4 to 11% (Table 1). Infected plants exhibited severe symptoms, including stunting, leaf chlorosis, wilting, and eventual plant death. At the stem base, yellow lesions were initially observed, which later developed into dry rot ranging from gray to dark brown, extending along the stem and containing embedded microsclerotia. Roots showed dry rot, necrosis, and dark lesions.

### 3.2. Morphological Analysis

**Figure 2:**
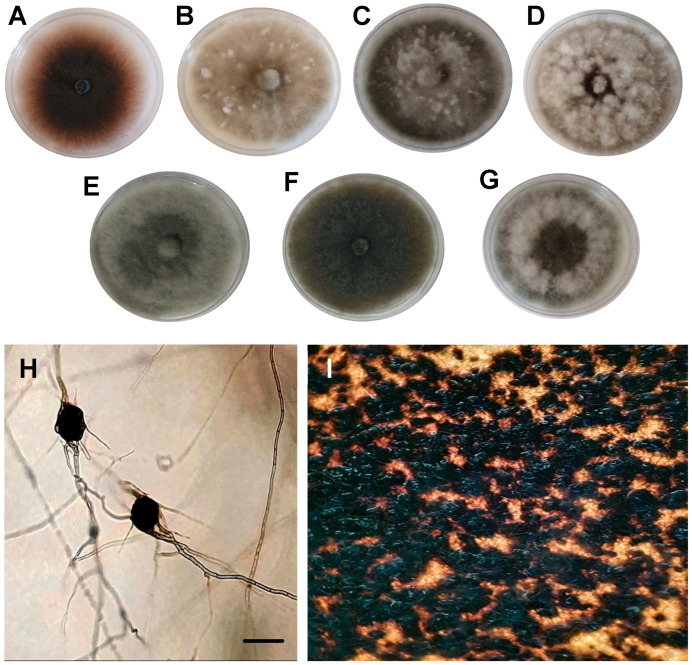
Colonies and survival structures of *Macrophomina phaseolina* grown on potato dextrose agar for six days, obtained from common bean plants showing charcoal rot symptoms. (**A**) Colony with flat mycelium and reddish-brown coloration. (**B**) Colony with abundant aerial mycelium, whitish to dark gray in color. (**C**) Colony with gray to black aerial mycelium. (**D**) Colony with abundant white aerial mycelium and a black center. (**E**) Colony with velvety mycelium, a black center, and an olive-black margin. (**F**) Colony with flat olive-black mycelium. (**G**) Colony with aerial mycelium showing alternating black and gray concentric rings. (**H**) Septate mycelium germinating from microsclerotia. (**I**) Abundant production of microsclerotia.

On PDA, colonies exhibited considerable variation in mycelial growth, and seven morphotypes were identified (Figure 2). Fifty percent of the isolates showed dense, floccose mycelial growth that was initially dark gray and later turned olive-black. Mycelial growth rate ranged from 26.03 to 60.47 mm day^−1^ (mean = 42.90 mm day^−1^). In colonies older than 5 days, microsclerotia developed that were spherical to irregular, black, smooth, and hard, measuring 55–165 µm in diameter (Figure 2). Septate mycelium and hyphae branching at approximately 90° angles were also observed.

### 3.3. Molecular Detection

**Figure 3:**
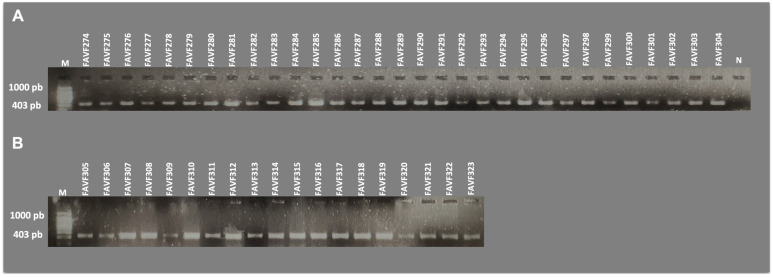
Molecular detection of *Macrophomina phaseolina* isolates using specific primers. (**A**,**B**) Agarose gels (1%) showing PCR amplification of the translation elongation factor 1-α (*tef1-α*) gene using specific primers (MpEFF/MpEFR). Lanes labeled “M” indicate the molecular weight marker, “N” indicates negative control, and the remaining lanes correspond to the amplification products of each of the 50 fungal isolates, in which a band of approximately 403 bp was observed. Species controls beyond the primer targets were not included.

Identification of *Macrophomina* isolates using species-specific primers targeting a fragment of the translation elongation factor 1-alpha (*tef1-α*) gene revealed that all 50 isolates corresponded to *Macrophomina phaseolina*. PCR amplification was obtained exclusively with the MpEFF/MpEFR primer pair specific for *M. phaseolina* (Figure 3), whereas no amplification was observed with the MsEFF/MsEFR and MeEFF/MeEFR primer pairs specific for *M. pseudophaseolina* and *M. euphorbiicola*, respectively.

### 3.4. Vegetative Compatibility

Mycelial compatibility tests of the 50 *M. phaseolina* isolates classified them into seven vegetative compatibility groups (VCGs), also referred to as hyphal fusion groups (Table 2). Except for VCG-V, which was found exclusively in fields located in the municipality of Ahome, and VCG-IV, which was detected only in Guasave, the remaining *M. phaseolina* VCGs showed no clear association with sampling location. Compatible and incompatible isolates were detected in fields from both Ahome and Guasave municipalities.

### 3.5. Pathogenicity and Aggressiveness

**Figure 4:**
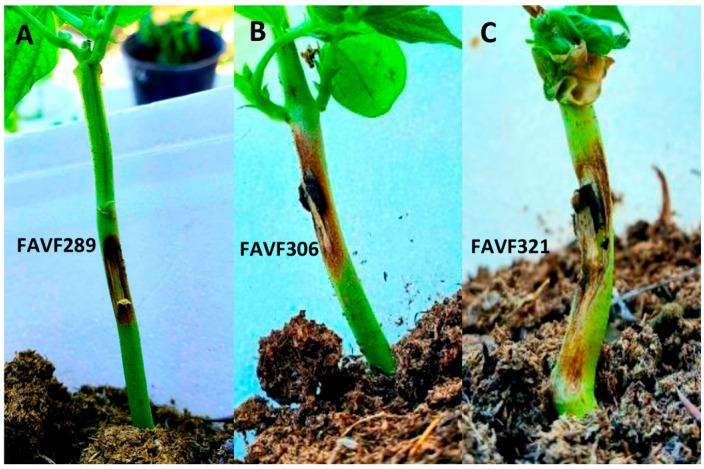
Thirty-day-old bean plants cv. Azufrado Higuera artificially inoculated with different isolates of M. phaseolina. (**A**) Necrotic lesion at the inoculation site caused by a low-virulence isolate. (**B**) Irregular dark brown lesion extending along the stem and induced by a moderately virulent isolate. (**C**) Dry rot and necrotic lesions extending along the entire stem, as well as wilting of branches and leaves caused by a highly virulent isolate.

All bean plants inoculated with *M. phaseolina* isolates developed necrotic lesions, stem base constriction, and dry rot along the stem seven days after inoculation (Figure 4). Subsequently, branch and leaf wilting were observed. Control plants remained asymptomatic, thereby confirming the pathogenicity of all 50 isolates. The cultural characteristics of the fungal colonies recovered from the artificially inoculated plants corresponded to the characteristics of the originally inoculated plants. Amplification with specific primers confirmed the identity of the isolates recovered from the inoculated plants.

**Figure 5:**
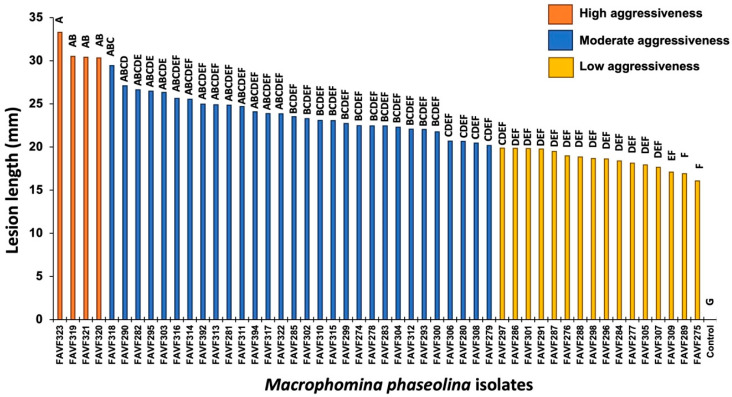
Lesion length (mm) caused by 50 *Macrophomina phaseolina* isolates associated with charcoal rot of common bean in northern Sinaloa, Mexico, measured 12 days after inoculation on stems of common bean cv. Azufrado Higuera. Columns sharing the same letter do not differ significantly according to the Tukey test (*p* ≥ 0.05).

Aggressiveness, measured as lesion length, was evaluated 12 days after inoculation and showed significant differences among isolates (*p* ≤ 0.05). The most aggressive isolates were FAVF323, FAVF319, FAVF321, and FAVF320, whereas the least aggressive were FAVF275, FAVF289, FAVF309, FAVF307, and FAVF305. Overall, 30% of the isolates exhibited low aggressiveness, 62% moderate aggressiveness, and 8% high aggressiveness (Figure 5).

## 4. Discussion

The recovery of *Macrophomina* isolates from all sampled fields demonstrates the widespread distribution of the pathogen in northern Sinaloa, Mexico, and highlights its potential epidemiological importance, particularly because this pathogen is favored by the water stress and high temperatures [4,5,7] prevalent in the study region. The incidence of charcoal rot caused by *Macrophomina phaseolina* across the 12 bean fields surveyed in this study averaged 8.3%, which is consistent with the 10% incidence reported for this disease in common bean in Kyrgyzstan [28]. However, incidences as high as 90% have been documented in specific localities, such as in Argentina [29]. Moreover, this pathogen may not only exhibit high field incidence but also be the most prevalent pathogen associated with root rot and wilt in beans, with *M. phaseolina* (26.7%) exceeding *Fusarium oxysporum* (13.6%) and *Agroathelia rolfsii* (5.6%) [30]. The relatively low incidence observed in northern Sinaloa (Mexico) may be attributed to seed treatment and agronomic practices, such as supplemental irrigation, despite local climatic and edaphic conditions that are conducive to disease development.

On PDA, *M. phaseolina* colonies exhibited considerable color and morphological diversity. Seven morphotypes were identified, and 50% of the isolates developed dense, floccose mycelial growth that was initially dark gray and later turned olive-black. Similar morphological diversity has been reported for *M. phaseolina* isolates obtained from cowpea [6]. In contrast, Leyva-Mir et al. (2015) [25] described only one colony type for *M. phaseolina* infecting sugarcane (*Saccharum officinarum* L.). The observed characteristics—spherical to irregular, black, smooth, hard microsclerotia measuring 60–165 µm, septate mycelium, and hyphae branching at approximately 90°—are consistent with previous reports [6,25,31]. The broad size range of microsclerotia reflects the morphological variability of this fungus, which can produce diverse colony forms and microsclerotia sizes [13,31,32,33].

Although multilocus phylogenetic analyses based on combined ITS, *act*, *tub2*, *cal*, and *tef1-α* sequences have been used to resolve cryptic species within *Macrophomina* (*M. phaseolina*, *M. pseudophaseolina*, and *M. euphorbiicola*) [6,11], the present study employed amplification of a fragment of the *tef1-α* gene using species-specific primers. The *tef1-α* gene contains sufficient phylogenetic signal and has been proposed as a primary marker for species discrimination within *Macrophomina* [34]. Molecular identification using species-specific primers targeting three cryptic species demonstrated that all 50 isolates corresponded to *M. phaseolina*, as amplification occurred exclusively with the primer pair specific to this species. These primers have been successfully applied to identify *Macrophomina* species causing diseases in several crops, including chickpea in México [21] and Italy [35], common bean in Italy [36], pigeonpea in India [37], cowpea in Ghana [38], watermelon in Brazil [39], and *Luffa* spp. in Brazil [40]. Therefore, the primers used in this study are sensitive and specific, providing results comparable to those of multilocus phylogenetic analyses while being more cost-effective, faster, and easier to implement. This approach enables rapid molecular identification of large isolate collections and reduces the financial burden associated with sequencing multiple loci [14]. The absence of amplification with primers specific for *M. pseudophaseolina* and *M. euphorbiicola* is noteworthy, as these species have recently been reported in other crops [11] and may coexist with *M. phaseolina*. Thus, the results confirm that *M. phaseolina* is the dominant species associated with charcoal rot of common bean in northern Sinaloa (Mexico), although the presence of other species cannot be entirely ruled out without broader sampling across other regions of Mexico, particularly given the extensive movement of propagative material.

The identification of seven vegetative compatibility groups (VCGs) suggests high genetic diversity within the evaluated *M. phaseolina* population. This finding is consistent with Cota-Barreras et al. (2022) [21], who identified six VCGs among 58 *M. phaseolina* isolates collected from commercial chickpea fields in Sinaloa (Mexico), concluding that substantial intraspecific variability exists within the same phylogenetic species. In contrast, Csöndes (2011) [31] reported high compatibility among 53 *M. phaseolina* isolates from sunflower (*Helianthus annuus*), maize (*Zea mays*), and soybean collected in Hungary, Spain, and Serbia, with only 24 incompatible pairings; geographically distant isolates were generally compatible. In the present study, except for VCG-V and VCG-IV, which were found exclusively in Ahome and Guasave, respectively, the remaining VCGs were distributed across divergent geographic points in both municipalities and readily fused during confrontation assays. These results indicate that VCG distribution cannot be directly correlated with geographic location, as identical or closely related genotypes may disperse over long distances. The transport of infected seed and plant material harboring fungal survival structures likely plays a key role in dissemination [31,41,42]. This finding is epidemiologically relevant because vegetative compatibility could favor DNA exchange via hyphal anastomosis between compatible isolates, thereby increasing the potential for parasexual recombination and maintaining genetic diversity in *M. phaseolina* [43]. The lack of a clear association between most VCGs and geographic origin suggests efficient pathogen dispersal, possibly mediated by infected seed, agricultural machinery, or movement of contaminated soil [5,44,45]. Nevertheless, the presence of exclusive VCGs in specific municipalities indicates that local population differentiation processes may also occur, influenced by management practices and environmental conditions. These findings have important epidemiological implications, as genetically diverse populations typically exhibit greater adaptive capacity [46], persistence, and potential to overcome management strategies, including the deployment of tolerant cultivars.

Molecular identification and pathogenicity assays, through consistent symptom reproduction, confirmed that *M. phaseolina* is the causal agent of charcoal rot of common bean in northern Sinaloa, Mexico. The rapid lesion development and disease progression are consistent with the pathogen’s necrotrophic lifestyle and its ability to colonize vascular and cortical tissues [47]. Furthermore, the pathogen showed no preference for a specific plant age, as it was isolated from both young and mature plants, in agreement with previous reports [3,6,11].

Aggressiveness data indicated that isolates with both low and high aggressiveness were present in the municipalities of Guasave and Ahome, suggesting that aggressiveness is not geographically structured. Significant variation in aggressiveness among isolates is a well-documented feature of *M. phaseolina* and has been associated with genetic differences, production of hydrolytic enzymes and toxins, and efficiency of host colonization [47,48]. The predominance of moderately aggressive isolates suggests a balanced population structure, whereas the presence of highly aggressive isolates, although limited, represents a relevant epidemiological risk.

Direct comparison of aggressiveness data with other studies was not feasible due to methodological variability in pathogenicity assays and the broad adaptive capacity and variability in aggressiveness of the pathogen [41,49,50,51]. Beas-Fernández et al. (2004) [52] suggested that pathogenicity may be associated with abundant microsclerotia production and that experimental conditions influence results. However, subsequent work by Beas-Fernández et al. (2006) [23] demonstrated that microsclerotial morphology and mycelial growth were not correlated with aggressiveness and that hyphal anastomosis among isolates was relatively low (24%). The most aggressive isolates were distributed among VCGs I, II, and VI, whereas the least aggressive isolates were assigned to VCGs I, II, and V, indicating no clear relationship between aggressiveness and vegetative compatibility. This suggests that isolates within the same VCG may potentially transfer virulence determinants through hyphal fusion, enabling less aggressive strains to acquire increased pathogenic potential. Similar lack of association among genotypes, aggressiveness, and host origin has been reported in other pathogen populations [44,53,54].

In this study, only *M. phaseolina* was identified infecting bean plants in commercial fields of Sinaloa, Mexico, consistent with previous reports [55] documenting this species in bean, maize, sesame, safflower, chickpea, soybean, guar, and peanut crops in northern Sinaloa. Although only *M. phaseolina* was detected, this finding is significant because this species has been reported to be more aggressive than other members of the genus [39,56,57]. Additionally, data on disease incidence, morphological diversity, and VCG distribution provide essential baseline information for future epidemiological and management studies in the region. The detected intraspecific variation likely reflects genetic and physiological differences among isolates, influencing their colonization capacity and response to environmental stress [7,54,58,59,60].

## 5. Conclusions

In summary, morphological, cultural, and molecular characterization using species-specific primers, for three *Macrophomina* species (*M. phaseolina*, *M. pseudophaseolina*, and *M. euphorbiicola*) of 50 fungal isolates obtained from bean plants with root and stem rot symptoms, confirmed that charcoal rot in northern Sinaloa (Mexico) is caused by *Macrophomina phaseolina*. Inoculation assays verified that all isolates were pathogenic and differed significantly in aggressiveness. Vegetative compatibility tests inferred hyphal anastomosis among certain isolates, resulting in seven mycelial compatibility groups among the 50 *M. phaseolina* isolates recovered from bean fields in Sinaloa, Mexico. *Macrophomina phaseolina* is a complex pathogen that requires further investigation to elucidate the genetic determinants underlying variation in aggressiveness, pathogen responses to environmental stress, and host varietal responses, and to develop sustainable management strategies for charcoal rot of common bean in Mexico.

## Figures and Tables

**Table 1:** Data of *Macrophomina phaseolina* isolates obtained from common bean plants exhibiting charcoal rot symptoms in commercial fields in northern Sinaloa, Mexico.

Isolate Code	Municipality	Collection Date	Field Number	Disease Incidence (%)	Coordinates
FAVF274, FAVF275, FAVF298, FAVF299	Ahome	November 2020	1	11.0	25°49′46.3″ N 108°51′10.5″ W
FAVF276, FAVF277 FAVF278, FAVF310	Guasave	November 2020	2	10.0	25°45′10.7″ N 108°47′48.7″ W
FAVF279, FAVF280, FAVF304	Guasave	November 2020	3	9.8	25°43′23.5″ N 108°46′53.4″ W
FAVF284, FAVF302, FAVF303, FAVF305	Guasave	December 2020	4	9.6	25°42′40.2″ N 108°47′54.4″ W
FAVF281, FAVF282, FAVF283	Guasave	December 2020	5	9.4	25°43′30.9″ N 108°49′35.9″ W
FAVF285, FAVF286, FAVF287, FAVF288, FAVF289, FAVF290, FAVF291, FAVF292	Guasave	December 2020	6	9.0	25°42′21.6″ N 108°50′20.8″ W
FAVF293, FAVF294, FAVF295, FAVF296, FAVF297	Ahome	December 2020	7	8.4	25°46′55.1″ N 108°52′19.2″ W
FAVF300, FAVF306, FAVF307, FAVF308, FAVF309	Ahome	December 2020	8	8.2	25°46′36.9″ N 108°51′10.5″ W
FAVF301	Ahome	January 2021	9	8.2	25°49′46.3″ N 108°56′58.1″ W
FAVF311, FAVF312, FAVF313, FAVF314, FAVF315	Ahome	January 2021	10	8.0	25°46′52.1″ N 108°52′7.5″ W
FAVF316, FAVF317, FAVF318	Guasave	January 2021	11	4.6	25°43′30.9″ N 108°49′35.9″ W
FAVF319, FAVF320, FAVF321, FAVF322, FAVF323	Ahome	January 2021	12	4.4	25°50′57.7″ N 108°59′8.6″ W

**Table 2:** Mycelial (vegetative) compatibility groups of *Macrophomina phaseolina* isolates obtained from common bean plants with charcoal rot symptoms in fields of northern Sinaloa, Mexico.

VCG	Number of Isolates	Isolate Numbers					Lesion Size (mm) *
I	15	FAVF274	FAVF275	FAVF277	FAVF278	FAVF284	22.06 BC
		FAVF286	FAVF288	FAVF293	FAVF296	FAVF312	
		FAVF313	FAVF316	FAVF321	FAVF322	FAVF323	
II	8	FAVF276	FAVF289	FAVF291	FAVF292	FAVF301	23.06 BC
		FAVF302	FAVF319	FAVF320			
III	4	FAVF285	FAVF304	FAVF307	FAVF308		20.90 AB
IV	4	FAVF279	FAVF280	FAVF281	FAVF300		21.85 AB
V	3	FAVF305	FAVF306	FAVF309			18.56 A
VI	7	FAVF282	FAVF283	FAVF290	FAVF294	FAVF297	23.46 BC
		FAVF298	FAVF299				
VII	9	FAVF287	FAVF295	FAVF303	FAVF310	FAVF311	25.47 C
		FAVF314	FAVF315	FAVF317	FAVF318		

VCG = vegetative compatibility groups. * Mean aggressiveness of isolates belonging to each VCG. Means sharing a common letter are not significantly different (*p* ≥ 0.05), according to Tukey’s test.

## Data Availability

The original contributions presented in this study are included in the article/Supplementary Material. Further inquiries can be directed to the corresponding author.

## Supplementary Materials

The following supporting information can be downloaded at: https://www.mdpi.com/article/10.3390/jof12030218/s1, Table S1: Metadata of the isolates from this study.
